# Discordance between mean glucose and time in range in relation to HbA_1c_ in individuals with type 1 diabetes: results from the GOLD and SILVER trials

**DOI:** 10.1007/s00125-024-06151-2

**Published:** 2024-04-26

**Authors:** Sofia Sterner Isaksson, Henrik Imberg, Irl B. Hirsch, Erik Schwarcz, Jarl Hellman, Magnus Wijkman, Jan Bolinder, Thomas Nyström, Helene Holmer, Sara Hallström, Arndís F. Ólafsdóttir, Sofia Pekkari, William Polonsky, Marcus Lind

**Affiliations:** 1 Department of Molecular and Clinical Medicine, Sahlgrenska Academy, University of Gothenburg, Gothenburg, Sweden; 2 Department of Medicine, NU Hospital Group, Uddevalla, Sweden; 3 Department of Mathematical Sciences, Chalmers University of Technology and University of Gothenburg, Gothenburg, Sweden; 4 Statistiska Konsultgruppen, Gothenburg, Sweden; 5 University of Washington, School of Medicine, Seattle, WA USA; 6 Department of Internal Medicine, Faculty of Medicine and Health, Örebro University, Örebro, Sweden; 7 Department of Medical Sciences, Uppsala University, Uppsala, Sweden; 8 Department of Internal Medicine and Department of Health, Medicine and Caring Sciences, Linköping University, Norrköping, Sweden; 9 Department of Medicine, Karolinska University Hospital Huddinge, Karolinska Institute, Stockholm, Sweden; 10 Department of Clinical Science and Education, Karolinska Institutet, Södersjukhuset, Stockholm, Sweden; 11 Department of Medicine, Centralsjukhuset, Kristianstad, Sweden; 12 Department of Medicine, Sahlgrenska University Hospital, Gothenburg, Sweden; 13 Behavioral Diabetes Institute, San Diego, CA USA

**Keywords:** Continuous glucose monitoring, HbA_1c_, Mean glucose, Time in range, Type 1 diabetes

## Abstract

**Aims/hypothesis:**

Previous studies have shown that individuals with similar mean glucose levels (MG) or percentage of time in range (TIR) may have different HbA_1c_ values. The aim of this study was to further elucidate how MG and TIR are associated with HbA_1c_.

**Methods:**

Data from the randomised clinical GOLD trial (*n*=144) and the follow-up SILVER trial (*n*=98) of adults with type 1 diabetes followed for 2.5 years were analysed. A total of 596 paired HbA_1c_/continuous glucose monitoring measurements were included. Linear mixed-effects models were used to account for intra-individual correlations in repeated-measures data.

**Results:**

In the GOLD trial, the mean age of the participants (± SD) was 44±13 years, 63 (44%) were female, and the mean HbA_1c_ (± SD) was 72±9.8 mmol/mol (8.7±0.9%). When correlating MG with HbA_1c_, MG explained 63% of the variation in HbA_1c_ (*r*=0.79, *p*<0.001). The variation in HbA_1c_ explained by MG increased to 88% (*r*=0.94, *p* value for improvement of fit <0.001) when accounting for person-to-person variation in the MG–HbA_1c_ relationship. Time below range (TBR; <3.9 mmol/l), time above range (TAR) level 2 (>13.9 mmol/l) and glycaemic variability had little or no effect on the association. For a given MG and TIR, the HbA_1c_ of 10% of individuals deviated by >8 mmol/mol (0.8%) from their estimated HbA_1c_ based on the overall association between MG and TIR with HbA_1c_. TBR and TAR level 2 significantly influenced the association between TIR and HbA_1c_. At a given TIR, each 1% increase in TBR was related to a 0.6 mmol/mol lower HbA_1c_ (95% CI 0.4, 0.9; *p*<0.001), and each 2% increase in TAR level 2 was related to a 0.4 mmol/mol higher HbA_1c_ (95% CI 0.1, 0.6; *p*=0.003). However, neither TIR, TBR nor TAR level 2 were significantly associated with HbA_1c_ when accounting for MG.

**Conclusions/interpretation:**

Inter-individual variations exist between MG and HbA_1c_, as well as between TIR and HbA_1c_, with clinically important deviations in relatively large groups of individuals with type 1 diabetes. These results may provide important information to both healthcare providers and individuals with diabetes in terms of prognosis and when making diabetes management decisions.

**Graphical Abstract:**

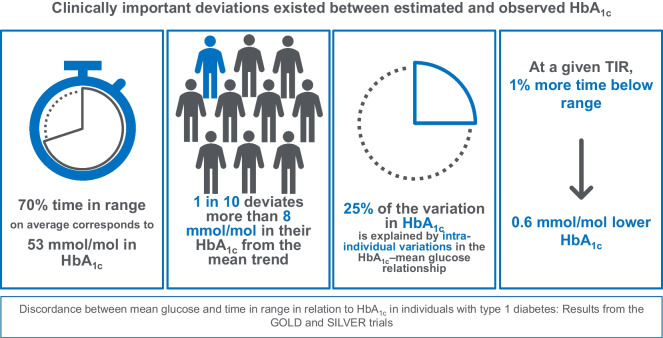

**Supplementary Information:**

The online version of this article (10.1007/s00125-024-06151-2) contains peer-reviewed but unedited supplementary material.


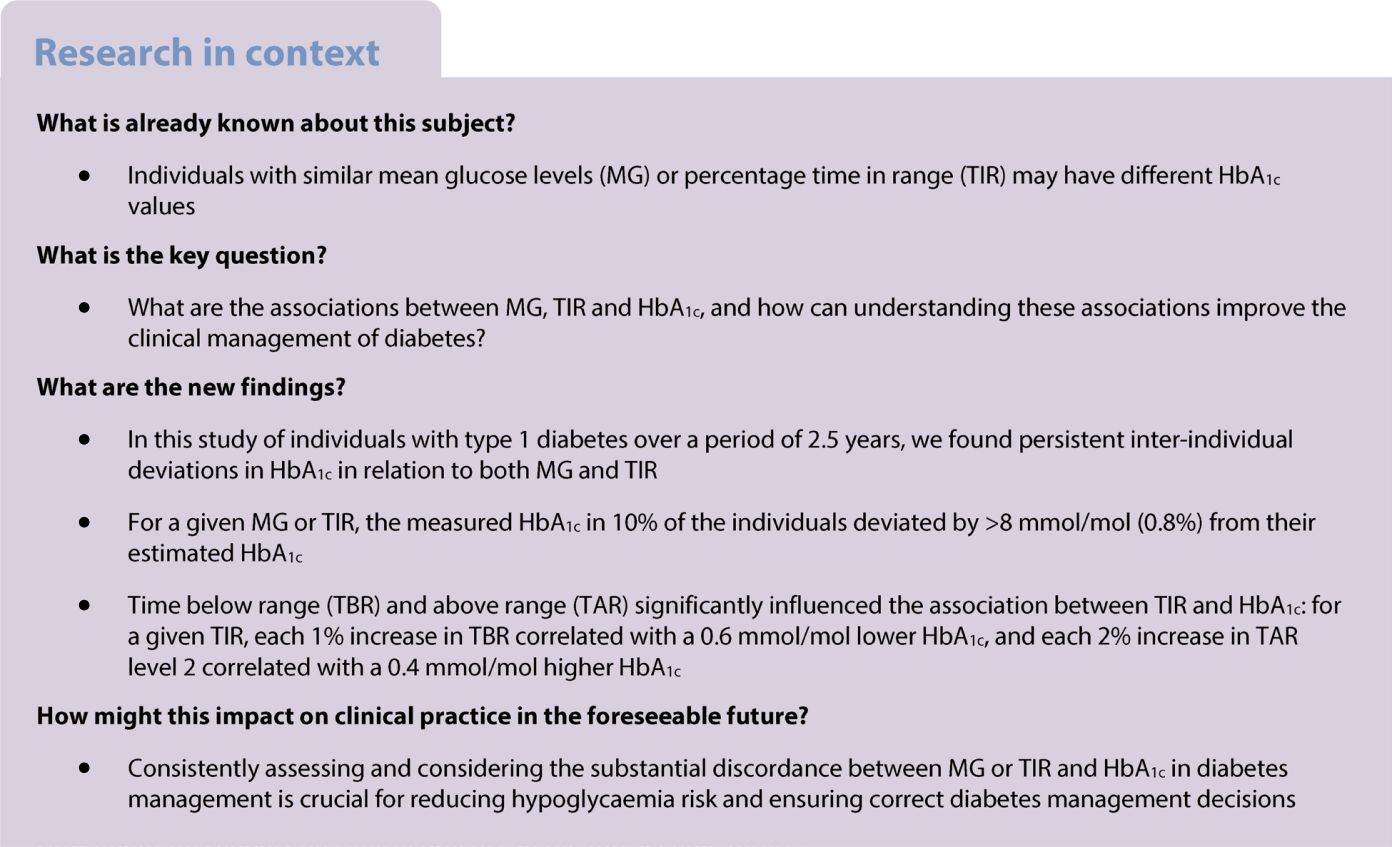


## Introduction

Glucose control is key to preventing diabetes complications in people with type 1 diabetes [1–4]. Analyses of glucose levels related to lower risk of diabetes complications have generally been based on the biomarker HbA_1c_ [2]. HbA_1c_ does not measure glucose level per se, but instead is based on glycation of haemoglobin and may be influenced by factors such as erythrocyte turnover and glycation rate [5, 6]. HbA_1c_ remains a key biomarker of complications in people with type 1 diabetes for several reasons. Landmark studies relating glucose control to complications have used HbA_1c_ as the metric of glucose control [1–4]. Large population-based studies following the prognosis of patients over long time periods have also relied on HbA_1c_ [3]. Furthermore, it is easy to measure and is a relatively cost-effective biomarker that is measured in most healthcare systems.

While HbA_1c_ generally remains the primary outcome for new indications of glucose-lowering treatments, many clinical judgements and research study endpoints are nowadays based on metrics obtained through continuous glucose monitoring (CGM) [7, 8]. This situation may be challenging for both individuals with diabetes and healthcare providers, as individuals may reach targets for certain metrics such as mean glucose (MG), time in range (TIR; % of time with glucose levels 3.9–10 mmol/l) or HbA_1c_ but not all of them. The TIR target of 70% has been set due to its relationship with an HbA_1c_ level of <53 mmol/mol (<7.0%) rather than data from long-term diabetes complication trials [7]. Therefore, it is important for clinicians to understand to what extent HbA_1c_ may differ in relation to both MG and TIR.

The treatment target for most adults with diabetes is an HbA_1c_ value or an MG-derived estimated HbA_1c_ glucose management indicator (GMI) [9] of <53 mmol/mol (<7.0%) [10], which corresponds to an MG of approximately 8.6 mmol/l (155 mg/dl). In clinical practice, questions may be raised when significant differences are observed between MG, TIR and HbA_1c_ if underlying explanatory factors such as anaemia could exist. Often such factors cannot be identified, complicating diabetes management for both individuals with diabetes and healthcare providers.

Genetic factors influencing the glycation rate of haemoglobin are probably important but are poorly understood and are not used in clinical practice. Deviations in glucose metrics are sometimes suspected to be due to insufficient CGM data being used to characterise overall glucose control. It is also speculative whether two individuals with the same MG but with different glucose patterns, such as long versus short periods with hypo- or hyperglycaemia, or high glycaemic variability versus stable glucose levels, will show different glycation rates and thereby different HbA_1c_, as suggested by others [11, 12]. Although earlier studies found a discordance between MG, TIR and HbA_1c_ [13–17], the associations are poorly understood.

The primary aim of the present study was to determine the associations between MG and HbA_1c_ using 2.5 years of data from the GOLD and SILVER trials, including whether different glucose patterns influence the relationship between MG and HbA_1c_. As a secondary aim, we also evaluated the associations between HbA_1c_ and TIR. The results are intended to create a basis for guiding patients, clinicians and researchers in the management of type 1 diabetes.

## Methods

### Design and participants

All analyses in the current study were performed using data from the GOLD trial (*n*=144) and the SILVER trial (*n*=98). The studies were approved by the ethics committee of University of Gothenburg, Sweden. All participants gave written informed consent, and the studies were registered on ClinicalTrials.gov (NCT02092051 and NCT02465411, respectively).

Briefly, the GOLD trial was a randomised crossover study comparing CGM use over 6 months versus self-monitoring of blood glucose over 6 months with a 4-month washout period in between [18]. Inclusion criteria were: adults with type 1 diabetes treated with multiple daily insulin injections, diabetes duration >1 year, fasting C-peptide level <0.3 nmol/l and with HbA_1c_ ≥58 mmol/mol (7.5%). Exclusion criteria were treatment with insulin pump. Full inclusion and exclusion criteria have been published elsewhere [19]. The primary endpoint was the difference in HbA_1c_ at the end of each treatment phase (total study period of 1.5 years). The SILVER trial was a follow-up study of the GOLD trial [20]. Participants who completed the GOLD trial were invited to participate in the SILVER trial extension, continuing CGM treatment for an additional year, with support from a diabetes nurse every third month. Participant-reported outcomes collected in both the GOLD and SILVER trials included the diabetes treatment satisfaction questionnaire (DTSQ), which measures aspects of treatment satisfaction [21, 22], and the hypoglycaemia confidence scale (HCS), which evaluates patient confidence in preventing and addressing hypoglycaemic events [23].

### Measurements

**Fig. 1:**
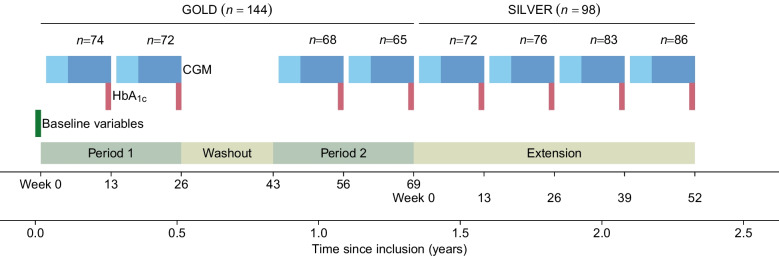
Flow chart of the study cohort during the GOLD and SILVER trials. CGM data obtained within 60 days (dark blue boxes) and 90 days (light blue boxes) from laboratory HbA_1c_ were used, corresponding to up to 30 days (main analysis) and 60 days (sensitivity analysis) of active CGM. At least 14 days of active CGM were required. Baseline variables (participant characteristics, participant-reported outcomes and laboratory measurements) were measured at the time of inclusion in the GOLD trial

The CGM systems used in the current study store up to 30 days of active CGM data. In the current analyses, CGM data from GOLD and SILVER trials comprising a minimum of 14 days of active CGM measurements within 60 days before laboratory HbA_1c_ were included. In the GOLD trial, all participants used the Dexcom G4 device (Dexcom, USA), but some participants switched to the Dexcom G5 device during the SILVER trial. The mean absolute relative difference for the Dexcom G4 device has been reported as 10.8±9.9% [24]. CGM data and HbA_1c_ measurements were collected after 13 and 26 weeks of CGM in the GOLD trial, and every 13th week for up to 52 weeks follow-up in the SILVER trial (Fig. 1). CGM data downloaded at week 4 were used to evaluate how extended CGM data affected analyses. HbA_1c_ was analysed according to the International Federation of Clinical Chemistry and Laboratory Medicine (IFCC) standard using a Variant II Turbo instrument (Bio-Rad Laboratories, USA). All blood samples were analysed at the Research Centre for Laboratory Medicine at Karolinska University Hospital, Stockholm, Sweden. Sex of participants was determined from the participants’ medical records.

### Primary analyses

The primary analysis focused on determining the association between MG and HbA_1c_ using paired MG and HbA_1c_ values from the GOLD and SILVER trials (Fig. 1). We investigated whether individual deviations in the MG–HbA_1c_ relationship persisted over time, indicating whether certain participants consistently deviated from the general MG–HbA_1c_ trend. Our main analyses were performed using data from the GOLD trial. Internal validation was used to evaluate whether such inter-individual deviations persisted over time, performing temporal validation using data from the SILVER trial. Additionally, we wished to determine whether clinically important differences between MG and HbA_1c_ existed. To do this, we estimated the magnitude of difference in HbA_1c_ among the 5% and 10% of individuals with the largest deviations in MG–HbA_1c_ from the general trend. Similar methods were applied for the secondary analysis relating HbA_1c_ to TIR.

### Exploratory analyses of potential explanatory factors

We hypothesised that certain glucose patterns, i.e. participants with the same MG but different glycaemic variability or time spent in hypoglycaemia, influenced HbA_1c_. We therefore explored whether various CGM metrics (time below range [TBR], TIR, time above range [TAR] level 2 and glycaemic variability) and the overall glucose distribution obtained through CGM explained deviations in MG–HbA_1c_ from the general trend in MG–HbA_1c_. Other possible explanatory factors investigated were age, sex, whether women were of fertile age (<50 years), diabetes duration, BMI, creatinine level, C-peptide level, C-reactive protein level, blood lipids, apolipoprotein levels and participant-reported outcomes (HCS and DTSQ scores). Baseline values for participant characteristics, participant-reported outcomes and laboratory measurements obtained at the time of inclusion in the GOLD trial were used in these analyses (Fig. 1).

Finally, we also assessed whether extended CGM profiles recorded at certain time points in the dataset (>30 days of active CGM data before HbA_1c_ measurement) influenced the MG–HbA_1c_ association. Additionally, we examined whether the impact of MG varied depending on whether it was measured during the daytime or at night.

Similar analyses were performed to assess potential explanatory factors for the relationship between HbA_1c_ and TIR. Analyses of the relationship between MG and TIR were also performed.

### Statistical analyses

Statistical analyses of the relationships between HbA_1c_ and MG or TIR were performed using linear mixed-effects models, with participant as random effect to account for individual deviations from the mean trend and intra-individual correlations in repeated-measures data. Individual predictions and individual trend lines were obtained from the best linear unbiased predictor of the random effects. Similar methods were used to study MG in relation to TIR and TBR.

Model fit was summarised using marginal and conditional *R*^2^ values and the marginal intraclass correlation coefficient (ICC) [25]. The marginal *R*^2^ is the fraction of variation in HbA_1c_ that may be explained by the mean trend, and hence is similar to the ordinary coefficient of determination. The marginal ICC is the fraction of variation in HbA_1c_ that may be explained by intra-individual variations around the mean trend. This is reported as the percentage improvement in *R*^2^ when accounting for person-to-person variations in the HbA_1c_–MG or HbA_1c_–TIR trend. The conditional (total) *R*^2^ is the sum of the marginal *R*^2^ and the marginal ICC, i.e. the total fraction of variation explained by the mean trend plus intra-individual variations. For comparability with previous studies, we also report the signed square root (*r*) of the marginal and conditional *R*^2^, which may be interpreted as the correlation according to the mean trend and the correlation when additionally accounting for intra-individual variations in the HbA_1c_–MG or HbA_1c_–TIR relationships. The improvement of fit between the marginal and conditional association was tested using a likelihood ratio test.

Multivariable analyses and interaction analyses were performed to investigate whether covariates explained individual variations in HbA_1c_ or altered the HbA_1c_–MG or HbA_1c_–TIR associations. A *p* value <0.05 in both the GOLD and SILVER trials was required for a finding to be considered statistically significant. Additionally, we investigated whether temporal factors (time of day or time since the glucose value was attained) or glucose patterns (i.e. the entire glucose distribution) affected the association with HbA_1c_. Additional details are provided in the electronic supplementary material (ESM Methods).

Statistical analyses were performed using SAS/STAT Software, Version 9.4 of the SAS System for Windows (SAS Institute, USA).

## Results

### Baseline characteristics

The baseline characteristics of participants from both trials included in the analyses are shown in Table 1. The mean age (± SD) among GOLD and SILVER trial participants was 44±13 and 46±13 years, respectively, with 63/144 (44%) and 39/98 (40%), respectively, being female. HbA_1c_ values were 72±9.8 (8.7±0.9%) and 71±8.0 mmol/mol (8.6±0.7%) at the start of the respective studies. HbA_1c_ values during the CGM periods in the GOLD and SILVER trials were 62.9±8.6 and 63.5±8.3 mmol/mol (7.9±0.8 and 8.0±0.8%), respectively. The corresponding MG and TIR values were 10.3±1.6 mmol/l and 48±14%, respectively, in the GOLD trial and 10.3±1.7 mmol/l and 49±15%, respectively, in the SILVER trial. In total, two participants (1%) in the GOLD trial had an eGFR <60 ml/min per 1.73 m^2^ and 20 (14%) had an albumin/creatinine ratio >3 mg/mmol. The median number of days for which CGM data at each pairwise HbA_1c_ value were available was 28.5 days (IQR 26.4–29.4) in the GOLD trial and 27.9 days (IQR 25.2–29.4) in the SILVER trial.

**Table 1:** Baseline characteristics of the study participants

Variable	GOLD trial population (*n*=144)	SILVER trial population (*n*=98)
Age, years	44±13	46±13
Female	63 (44)	39 (40)
Time from diabetes onset to inclusion, years	23±12	24±12
BMI, kg/m^2^	27±4.4	27±4.6
Smoking status		
Current	18 (13)	7 (7)
Previous	32 (22)	26 (27)
Never	94 (65)	65 (66)
HbA_1c_, mmol/mol	72±9.8	71±8.0
Creatinine, µmol/l	71±14.3	72±14.9
eGFR, ml/min per 1.73m^2^	107 (94–117)	106 (93–117)
eGFR <60 ml/min per 1.73m^2^	2 (1)	1 (1)
ACR, mg/mmol	0.8 (0.4–2.1)	0.8 (0.4–2.1)
ACR >3 mg/mmol	20 (14)	15 (15)
Total cholesterol, mmol/l	4.5±0.9	4.5±0.9
LDL, mmol/l	2.5±0.8	2.4±0.5
HDL, mmol/l	1.6±0.5	1.6±0.5
Triacylglycerols, mmol/l	0.9±0.6	0.9±0.5
Apolipoprotein A1, mg/ml	1.7±0.3	1.6±0.4
Apolipoprotein B, mg/ml	0.9±0.2	0.9±0.2
CRP, mg/l	3.0±5.5	3.3±6.6

Continuous variables are reported as mean ± SD or median (IQR). Categorical variables are reported as *n* (%)

All participants were white

ACR, albumin/creatinine ratio; CRP, C-reactive protein

### Primary analysis: HbA_1c_ in relation to MG

**Fig. 2:**
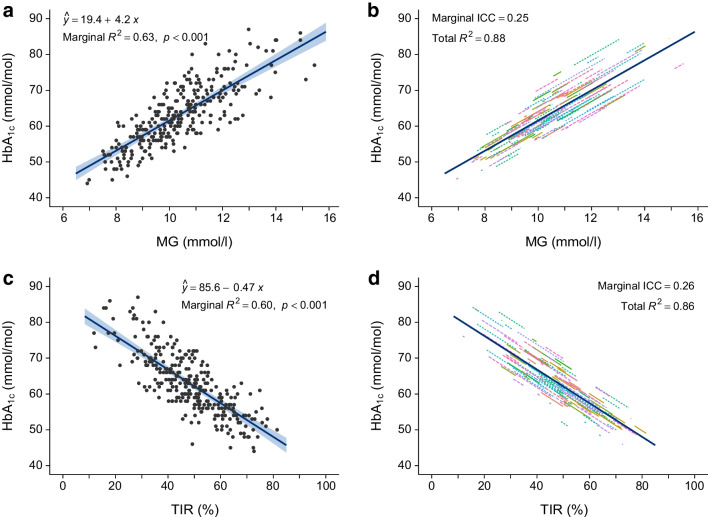
Relationships between HbA_1c_ and MG (**a**, **b**) and between HbA_1c_ and TIR (3.9–10.0 mmol/l) (**c**, **d**) for data from the GOLD trial. (**a**, **c**) Mean trend. (**b**, **d**) Individual trend lines. Statistical analyses were performed using linear mixed-effects models. The mean trend and individual trend lines were derived from the same model. Total *R*^2^ = marginal *R*^2^ + marginal ICC

For the primary analysis, MG explained 63% of the variation in HbA_1c_ in the GOLD trial (*r*=0.79, *p*<0.001). Differences in person-to-person variation in the relationship between MG and HbA_1c_ explained an additional 25% of the variation in HbA_1c_ (*p*<0.001) (Fig. 2). Thus, MG together with inter-individual effects explained 88% of the variation in HbA_1c_ in the GOLD trial (*r*=0.94). Inter-individual deviations persisted over time, with a coefficient of determination (*R*^2^) of 78% (*r*=0.88) when individual predictions from this model were evaluated prospectively using data from the SILVER trial. For a given MG, the HbA_1c_ values in 5% and 10% of the individuals deviated more than 9.9 and 8.3 mmol/mol (0.9 and 0.8%) from the mean trend, respectively.

### Secondary analysis: HbA_1c_ in relation to TIR

In the secondary analysis relating TIR to HbA_1c_, TIR explained 60% (*r*=−0.77) of the variation in HbA_1c_ in the GOLD trial dataset, which increased to 86% (*r*=−0.93) when additionally accounting for person-to-person variation in the relationship between TIR and HbA_1c_ (*p*<0.001) (Fig. 2). A TIR of 70% corresponded to an HbA_1c_ of 53 mmol/mol (7.0%). For a given TIR, the HbA_1c_ values in 5% and 10% of the individuals deviated more than 9.9 and 8.3 mmol/mol (0.9 and 0.8%) from the mean trend, respectively.

### Explanatory factors for the HbA_1c_–MG relationship

No CGM metrics (TBR, TIR, TAR or glycaemic variability), nor the glucose distribution based on CGM, influenced the association between MG and HbA_1c_ persistently in the GOLD and SILVER trials (ESM Tables 1 and 2, ESM Figs 1 and 2). Other exploratory variables, including age, sex, renal function, BMI, C-peptide level, blood lipids, apolipoproteins, HCS or DTSQ score, showed no persistent association in the GOLD and SILVER trials.

Using extended time periods of CGM data during weeks 0–13 had little influence on the correlation between MG and HbA_1c_. The correlation was 0.79 using 30 days of active CGM (up to 60 days before HbA_1c_ measurement) and 0.80 using 60 days of active CGM (up to 90 days before HbA_1c_ measurement). Applying unequal weights depending on the time since the glucose value was attained when estimating MG did not result in an improved correlation (*p*=0.70 for improvement of fit)*.* There was also no significant improvement when daytime and night-time glucose values were weighted unequally when assessing the relationship between MG and HbA_1c_ (*p*=0.18).

### Explanatory factors for the HbA_1c_–TIR relationship

**Fig. 3:**
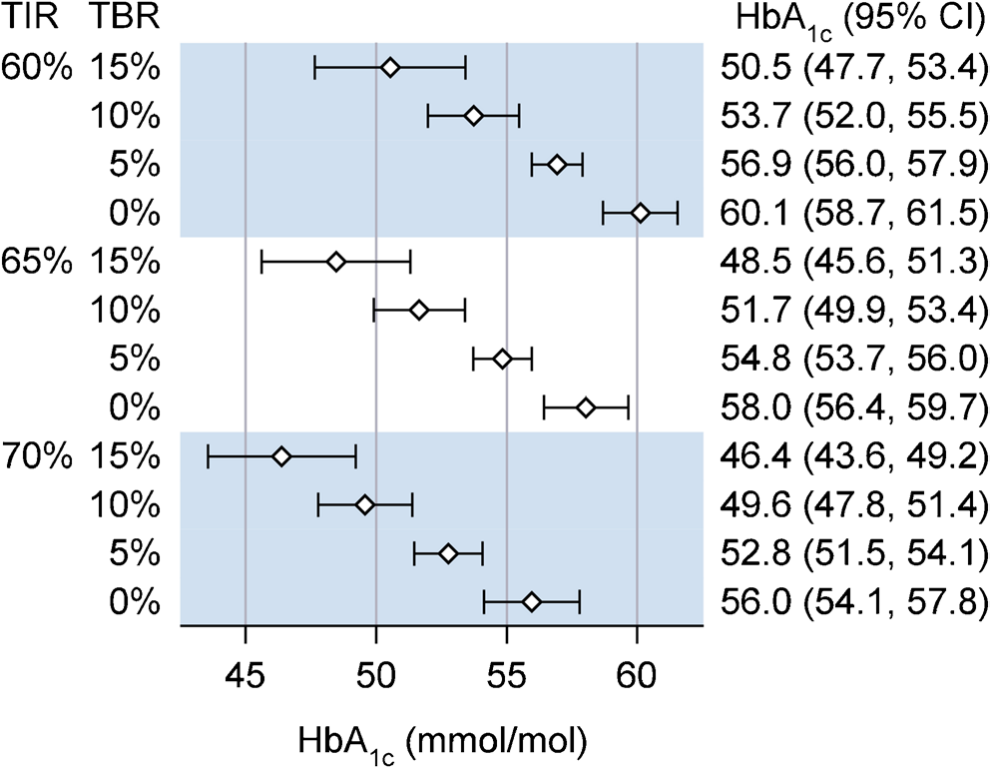
Estimated HbA_1c_ at a given level of TIR (3.9–10.0 mmol/l) and TBR (<3.9 mmol/l)

There was a significant association for TBR (<3.9 mmol/l) (*p*<0.001 in the GOLD trial; *p*=0.012 in the SILVER trial) and TAR level 2 (>13.9 mmol/l) (*p*=0.003 in the GOLD trial; *p*=0.007 in the SILVER trial) in terms of explaining deviations in HbA_1c_ from the estimated HbA_1c_–TIR mean trend (ESM Tables 3 and 4). At a given TIR, each 1% increase in TBR was related to a 0.6 mmol/mol lower HbA_1c_ (95% CI 0.4, 0.9; *p*<0.001) and each 2% increase in TAR level 2 was related to a 0.4 mmol/mol higher HbA_1c_ (95% CI 0.1, 0.6; *p*=0.003). Figure 3 shows the impact of TBR on the association between TIR and HbA_1c_. No other CGM metric or variable influenced the association between TIR and HbA_1c_ when adjusting for TBR and TAR (ESM Tables 3 and 4). When adjusting for the MG, neither TIR, TBR nor TAR level 2 were significantly related to HbA_1c_ (ESM Table 1).

### Associations between MG and TIR

For a given TIR, MG decreased by 0.6 mmol/l (95% CI 0.5, 0.7) per 5% increase in TBR. The association between MG and TIR is shown in ESM Fig. 3. A TIR of 70% with TBR of 0% vs 15% corresponded to an MG of 8.5 vs 6.8 mmol/l.

## Discussion

### Principal findings

In this study, based on data from the GOLD and SILVER trials, we found important inter-individual deviations in HbA_1c_ in relation to both MG and TIR that persisted over a combined 2.5-year follow-up period. These inter-individual deviations were of clear clinical importance, with notable deviations in HbA_1c_ (>8 mmol/mol, >0.8%) observed in 10% of the individuals. The relationship was similar for men and women and glucose patterns had minimal or no impact on the association between MG and HbA_1c_. However, TBR had additional intra- and inter-individual influences on the association between TIR and HbA_1c_. At a given TIR, each 1% increase in TBR corresponded to a 0.6 mmol/mol lower HbA_1c_, and each 2% increase in TAR level 2 to a 0.4 mmol/mol higher HbA_1c_.

### Previous studies

Previous studies relating MG to HbA_1c_ found correlation coefficients of *r*=0.78–0.80 and 0.73 [13–15, 17], corresponding to our findings of *r*=0.79. However, we found that, when taking into account differences in systematic person-to-person variations of MG relative to HbA_1c_, the correlation increased to *r*=0.94. A TIR of 70% has previously been related to an HbA_1c_ of <53 mmol/mol (<7%) [13, 14], and is commonly used in clinical practice and research as a basis for judging low complication risk based on TIR [10]. In the current study, a TIR of 70% was, on average, also related to an HbA_1c_ of 53 mmol/mol (7%), but the HbA_1c_ deviated systematically over time by more than 8 mmol/mol (0.8%) for over 10% of the individuals. Our findings of an influence of TBR on HbA_1c_ in relation to TIR but not in relation to MG is novel, and this question have not been extensively studied in previous research.

### Explanations and interpretations

HbA_1c_ represents the glycation rate of haemoglobin, and is thus dependent on erythrocyte turnover and the lifespan of erythrocytes (approximately 120 days) [26]. One potential explanation for deviations in the relationship between MG or TIR and HbA_1c_ may be due to incomplete glucose data over time. However, consistent with previous results [27], longer measurement periods for MG did not show significantly stronger associations with HbA_1c_. One possible explanation may be that the most recent periods have a relatively greater influence on the HbA_1c_ [26], and that individuals generally have a relatively stable MG over time [28]. We also speculated that the glycation rate may not solely be explained by the MG but also by other characteristics of the distribution, such as fluctuations or extended periods with hypoglycaemia, as suggested previously [11, 12]. However, various glucose patterns present at the same MG did not explain deviations between MG and HbA_1c_, and nor did glycaemic variability.

Anaemia, which leads to a shorter erythrocyte lifespan (e.g. through haemolysis), can influence the HbA_1c_, but in the current study, women of fertile age, who are more commonly prone to anaemia, did not deviate in their association of MG or TIR with HbA_1c_.

Although race may influence the association of MG and TIR with HbA_1c_ [29, 30], it was not a factor in the current study, in which all participants were white. Impaired renal function can affect HbA_1c_ [31], but few individuals in the GOLD trial had impaired renal function. Instead, it seems plausible that genetic rather than glucose-related factors that influence glucose transport into erythrocytes and the glycation rate of the haemoglobin explain inter-individual differences for high and low glycators (as defined below) [32]. In addition, differences in TBR at a given TIR correspond to various MG values and thereby explain differences in HbA_1c_ for a single individual over time.

### Clinical implications

There is a critical need for clinicians to be aware of the association between MG and HbA_1c_. Values for these glucose indices are typically presented to individuals with type 1 diabetes during clinical visits, but they can also get information about their calculated GMI through CGM system-generated ambulatory glucose profile reports [33]. We propose that clinicians should assess both HbA_1c_ and GMI, and not only acknowledge if a difference exists, but also record its magnitude and direction accurately. Repeated deviations between HbA_1c_ and GMI in the same direction will suggest whether an individual is a high or low glycator [32]. A high glycator is indicated when HbA_1c_ is consistently higher than GMI, and vice versa for a low glycator. Large discordances between MG and HbA_1c_ may influence diabetes management [32]. From a global perspective, CGM is not available to most people with type 1 diabetes. When possible, temporary use of CGM will be valuable to confirm the true MG and whether major discordances with HbA_1c_ exist.

Although insulin dosing per se is based on CGM or capillary glucose levels, it has been proposed that individuals with a low MG but high HbA_1c_ may be at increased risk of hypoglycaemia [34]. Individuals with diabetes are generally aware of HbA_1c_ targets, as this information is repeatedly given to them by clinicians at clinical visits. Hence, there is a risk that some individuals may strive for intensified treatment if HbA_1c_ is high when GMI is on target, especially if healthcare providers do not inform the individual of discordances between the two [34]. Moreover, individuals may experience increased anxiety regarding the risk of complications correlating with a higher HbA_1c_ [35]. In contrast, on-target HbA_1c_ but high MG may lead to insufficient intensification of treatment [34]. However, HbA_1c_ is still of primary focus in clinical practice and is also used for quality assessment between clinics and countries [36, 37].

TIR has increasingly come into greater focus in clinical practice and research over time [7, 8]. As HbA_1c_ differs in relation to MG, it is possible that it will also differ in relation to TIR, as TIR is closely related to MG at a certain TBR. As the target TIR of 70% was established based on its overall relationship with an HbA_1c_ of <53 mmol/mol (7.0%), clinicians need to be aware, as discussed earlier in the context of the MG–HbA_1c_ relationship, that discordances between HbA_1c_ and TIR for individuals must be recognised and considered in diabetes management. Moreover, for the same individual, a specific TIR for an individual with greater TBR will intuitively relate to lower HbA_1c_ due to lower MG, as confirmed in the current study. Hence, HbA_1c_ and GMI may shift over time while maintaining a stable TIR if, for example, adjustments in diabetes care are made that alter the magnitude of TBR or TAR.

At present, it is not known which glucose index (HbA_1c_, TIR or MG/GMI) is the most effective indicator for diabetes complications. While it may seem reasonable that MG per se would be the most predictive, HbA_1c_ is considered a marker for the glycation rate and glycation end-products, which are related to complications beyond its relationship with MG [38, 39]. Additionally, some studies have shown associations between TIR and complications [40, 41]. Long-term studies, preferably following participants from the time of diagnosis (as profound legacy effects exist from previous hyperglycaemic episodes [4]), are necessary but take time to perform. An international standardisation for CGM systems of their calibration to blood is also crucial, as CGM systems have been shown to systematically deviate from blood glucose values, which can influence CGM metrics [42].

It is likely that HbA_1c_, TIR and MG/GMI will remain as essential complementary metrics. Thus, it is crucial that clinicians assess and communicate these metrics effectively to individuals with diabetes in an appropriate way to reduce complication risk and decrease diabetes-related distress.

### Strengths and limitations

A major strength of the current study is that, in contrast to most earlier studies, participants were followed over 2.5 years using CGM devices from the same manufacturer and HbA_1c_ was centrally analysed. Measurements of HbA_1c_ and CGM metrics were obtained at similar time points. This is of critical importance when considering possible systematic deviations of CGM-based metrics over time in relation to HbA_1c_. A limitation is that we did not obtain data on anaemia and blood disorders, which are factors that could possibly affect HbA_1c_. However, adjusting for women of fertile age, who are known to have anaemia more commonly, did not influence the associations. All participants were white, used multiple daily insulin injections for insulin delivery, with HbA_1c_ >58 mmol/mol (7.5%), and had overall good renal function, and the results may be limited to this population. Information on socioeconomic factors was not collected and the influence of such factors could therefore not be evaluated. We tested multiple variables to elucidate the relationship between MG and HbA_1c_. Our focus was primarily on CGM metrics, as we considered them to be more plausible explanatory factors. While performing multiple tests may increase the risk of false-positive results, this risk was mitigated by requiring positive findings in both the GOLD and SILVER trials. Although it should be acknowledged that control of the potential type 1 error rate was not strict, all variables evaluated as potential explanatory factors were judged to be non-significant except TAR and TBR for the association of TIR with HbA_1c_. The significance of these factors has a plausible explanation as greater TBR (TAR) at a given TIR leads to a lower (higher) MG, and hence a lower (higher) HbA_1c_.

### Conclusions

In conclusion, the same HbA_1c_ value may be observed in people with significantly different MG/GMI or TIR. This information is crucial for both healthcare providers and individuals with diabetes when making diabetes management decisions. Consequently, MG/GMI (obtained from including significant periods of CGM data) should be evaluated at clinical visits to determine whether people with type 1 diabetes have an HbA_1c_ that is higher or lower than the mean trend. Additionally, time spent in hypoglycaemia should always be considered together with TIR. The evaluation of MG/GMI and HbA_1c_, with minimal time spent in hypoglycaemia, should be a primary focus in clinical practice to achieve glucose control with a low risk of both acute and long-term complications.

### Supplementary Information

Below is the link to the electronic supplementary material.

Supplementary file1 (PDF 415 KB)

## Data Availability

The data that support the findings of this study are not openly available. They are available from the corresponding author upon reasonable request.

## Abbreviations

CGM: Continuous glucose monitoring
DTSQ: Diabetes treatment satisfaction questionnaire
GMI: Glucose management indicator
HCS: Hypoglycaemia confidence scale
ICC: Intraclass correlation coefficient
MG: Mean glucose level
TAR: Time above range
TBR: Time below range
TIR: Time in range

## Authors’ relationships and activities

ES serves on advisory boards for Abbott and Sanofi, and has received lecture payments from Sanofi, Boehringer Ingelheim, Lilly and Novo Nordisk. IBH has received research grants from Dexcom, and received honoraria or fees for consulting with Abbott Diabetes Care, Hagar and embecta. JB has received honoraria for consulting and/or lecture fees from Abbott Diabetes Care, the MannKind Corporation, Nanexa, Nordic InfuCare, Novo Nordisk and Sanofi. JH serves or has served on advisory boards for Sanofi, Eli Lilly, Novo Nordisk, Boehringer Ingelheim and Abbott, and has received lecture payments from Sanofi, Boehringer Ingelheim, Rubin Medical, Nordic Infucare, Bayer, Amgen, Eli Lilly and Novo Nordisk. ML has received research grants from Eli Lilly and Novo Nordisk and been a consultant or received honoraria from Astra Zeneca, Boehringer Ingelheim, Nordic Infucare, Eli Lilly and Novo Nordisk. MW has served on advisory boards or lectured for MSD, Lilly, Novo Nordisk and Sanofi, and has organised a professional regional meeting sponsored by Eli Lilly, Rubin Medical, Sanofi, Novartis and Novo Nordisk. SH has lectured for Novo Nordisk. TN has received unrestricted grants from AstraZeneca and Novo Nordisk, and has served on national advisory boards for Amgen, Abbot, Novo Nordisk, Sanofi-Aventis, Eli Lilly, MSD and Boehringer Ingelheim. WP has received research support from Dexcom and Abbott Diabetes Care, and has received honoraria for consulting from Dexcom and Abbott Diabetes Care. The remaining authors declare that there are no relationships or activities that might bias, or be perceived to bias, their work.
