# Supplementary material for: Discordance between mean glucose and time in range in relation to HbA1c in individuals with type 1 diabetes: results from the GOLD and SILVER trials
Source: Diabetologia. 2024 Apr 26;67(8):1517–26. doi: 10.1007/s00125-024-06151-2 (PMC11343832; doi:10.1007/s00125-024-06151-2)

Electronic supplementary material (ESM)

This appendix has been provided by the authors to give readers additional information about their work.

Supplement to:

Discordance between mean glucose and time in range in relation to HbA_{1c} in individuals with type 1 diabetes: results from the GOLD and SILVER trials

Authors: Sofia Sterner Isaksson, Henrik Imberg, Irl B Hirsch, Erik Schwarcz, Jarl Hellman, Magnus Wijkman, Jan Bolinder, Thomas Nyström, Helene Holmer, Sara Hallström, Arndís F Ólafsdóttir, Sofia Pekkari, William Polonsky, Marcus Lind.

Table of contents

ESM Methods: Additional statistical analysis details……………………….…………………..3

ESM Table 1. Linear mixed-effects regression models relating HbA_{1c} to mean glucose, glycaemic variability measures, time in glycaemic ranges, patient characteristics, and baseline covariates…………………………………………………………………………..5

ESM Table 2. Interaction analyses relating HbA_{1c} to mean glucose, glycaemic variability measures, time in glycaemic ranges, patient characteristics, and baseline covariates…..……7

ESM Table 3. Linear mixed-effects regression models relating HbA_{1c} to time in range, glycaemic variability measures, time in glycaemic ranges, patient characteristics, and baseline covariates…..9

ESM Table 4. Interaction analyses relating HbA_{1c} to time in range, glycaemic variability measures, time in glycaemic ranges, patient characteristics, and baseline covariates……….11

ESM Figure 1. Non-linear association between HbA_{1c} and the distribution of glucose values derived on data from the GOLD study.…………………..………………..................……..13

ESM Figure 2. Non-linear association between HbA_{1c} and the distribution of glucose values validated on data from the SILVER study…......................……….…..………………...……14

ESM Figure 3. Estimated mean glucose at a given level of TIR and TBR ………………...15

ESM Methods: Additional statistical analysis details

We evaluated possible time-dependent and non-linear and associations between blood glucose and HbA_{1c} by correlating HbA_{1c} to a weighted mean glucose metric depending on i) time of day, ii) time since the glucose value was attained, and iii) actual glucose value, as further detailed below.

Time of the day

The weighted mean glucose metric was calculated as

$$wMG = w * MG_{daytime} + (1 - w)MG_{nighttime},$$

where $MG_{daytime}$ is the mean glucose during daytime (06:00–22:00), $MG_{nighttime}$ the mean glucose during nighttime (22:00–06:00), and $w \in [0, 1]$ a weight parameter to be estimated. The optimal weight $\hat{w}$ was estimated by maximising the log-likelihood of the random intercepts model

$$HbA1c_{ij} = \beta_0 + \beta_1 * wMG_{ij} + b_i + \varepsilon_{ij}, \quad (1)$$

where $HbA1c_{ij}$ is the observed HbA_{1c} for individual i at visit j, wMG_{ij} the corresponding weighted mean glucose metric, b_i a subject-specific random intercept, and ε_{ij} a random (residual) error. The random intercepts and residual errors were assumed to be uncorrelated and normally distributed with zero mean. The null hypothesis that $w = 2/3$, i.e., that measurements obtained during daytime and nighttime contribute equally to the association with HbA_{1c} ($w = 2/3 \Leftrightarrow$ 16 hours daytime divided by 24), was tested using a likelihood ratio test.

Similar calculations were performed for time in range.

Time since the glucose value was attained

The weighted mean glucose metric was calculated as

$$wMG = \sum_t w(t)x(t),$$

where $w(t) = Ce^{-kt}$ is an exponential weight function, t the time since the glucose value was attained, $x(t)$ the observed glucose value at timepoint t, and k an exponential decay parameter to be estimated. The constant C was chosen for each subject and visit so that $\sum_t w(t) = 1$, with summation over all timepoints for which a glucose measurement was available. The optimal parameter $\hat{k}$ was estimated by maximising the log-likelihood of the random intercepts model (1) as a function of the coefficient k. The null hypothesis that $k = 0$, i.e., that all glucose measurements contribute equally to the association with HbA_{1c}, was tested using a likelihood ratio test.

Similar calculations were performed for time in range.

Distribution of glucose values

The weighted mean glucose metric was calculated as

$$wMG = \int_{2.22}^{22.2} \alpha(x) dP(x),$$

where 2.22 and 22.2 is the lower and upper measurement limit of the CGM device, x the glucose value, $\alpha(x)$ a weight function, and $P(x)$ a probability measure for the distribution of glucose values. The probability measure $P(x)$ was taken as the empirical measure, i.e., the relative frequency of glucose values. Values <2.22 mmol/l were taken as 2.22 mmol/l and values ≥22.2 mmol/l taken as 22.2 mmol/l. The weight function $\alpha(x)$ was modelled using fractional polynomials, i.e., as a function on the form

$$\alpha(x) = \alpha_0 + \alpha_1 x^{-2} + \alpha_2 x^{-1} + \alpha_3 x^{-0.5} + \alpha_4 \log(x) +$$

$$\alpha_5 x^{0.5} + \alpha_6 x + \alpha_7 x^2 + \alpha_8 x^3. \qquad (2)$$

To avoid overfitting, some of the coefficients α_j were set to zero. The best fitting fractional polynomial for the random intercepts model (1) was selected using best subset selection of all fractional polynomials including at most three terms by minimising the Akaike Information Criterion (AIC).

Combining (1) and (2), the model form the mean HbA_{1c} related to the distribution of blood glucose can also be written on the form

$$\widehat{HbA_{1c}} = \int \beta(x) dP(x).$$

We note that a linear model corresponds to a linear relationship between mean glucose and HbA_{1c}.

ESM Table 1. Linear mixed-effects regression models relating HbA_{1c} (mmol/mol) to mean glucose (mmol/l), glycaemic variability measures, time in glycaemic ranges, patient characteristics, and baseline covariates. Results are presented as regression coefficients with 95% confidence intervals.

	GOLD study			SILVER study		
**Covariate**	**Intercept**	**Mean glucose**	**Covariate coefficient**	**Intercept**	**Mean glucose**	**Covariate coefficient**
**CGM SD (mmol/l)**	20.20 (15.44, 24.96)	4.39 (3.83, 4.94)	-0.66 (-1.99, 0.67) p=0.33	21.49 (17.29, 25.69)	4.08 (3.62, 4.55)	-0.06 (-1.18, 1.05) p=0.91
**CGM CV (%)**	23.74 (15.67, 31.81)	4.12 (3.67, 4.57)	-9.00 (-23.03, 5.02) p=0.21	21.02 (14.31, 27.72)	4.07 (3.68, 4.47)	0.85 (-10.75, 12.45) p=0.89
**MAGE (mmol/l)**	20.45 (15.49, 25.40)	4.36 (3.83, 4.88)	-0.28 (-0.85, 0.29) p=0.34	21.73 (17.57, 25.89)	4.11 (3.70, 4.52)	-0.09 (-0.42, 0.25) p=0.60
**Percentage of time below range (<3.9 mmol/l)**	22.17 (16.20, 28.14)	4.01 (3.49, 4.53)	-0.20 (-0.48, 0.09) p=0.17	19.15 (14.24, 24.05)	4.23 (3.80, 4.66)	0.19 (-0.05, 0.43) p=0.12
**Percentage of time in range (3.9–10.0 mmol/l)**	30.87 (9.35, 52.39)	3.51 (2.14, 4.88)	-0.09 (-0.24, 0.07) p=0.28	40.53 (22.83, 58.24)	2.88 (1.74, 4.01)	-0.14 (-0.27, -0.01) p=0.030
**Percentage of time in target (3.5–7.8 mmol/l)**	27.31 (12.01, 42.62)	3.68 (2.60, 4.76)	-0.08 (-0.23, 0.07) p=0.29	31.67 (19.69, 43.66)	3.37 (2.52, 4.22)	-0.11 (-0.22, 0.01) p=0.075
**Percentage of time above range level 2 (>13.9 mmol/l)**	13.32 (1.10, 25.54)	5.00 (3.47, 6.53)	-0.10 (-0.30, 0.09) p=0.29	17.11 (6.83, 27.40)	4.61 (3.35, 5.87)	-0.07 (-0.22, 0.08) p=0.37
**Female**	19.43 (14.92, 23.93)	4.20 (3.77, 4.63)	0.30 (-1.27, 1.86) p=0.71	21.13 (17.17, 25.09)	4.03 (3.66, 4.41)	1.66 (-0.06, 3.39) p=0.058
**Age (years)**	18.21 (12.70, 23.73)	4.23 (3.80, 4.66)	0.02 (-0.04, 0.08) p=0.45	20.18 (15.03, 25.33)	4.08 (3.70, 4.45)	0.02 (-0.04, 0.09) p=0.46
**Female <50 years**	19.22 (14.72, 23.73)	4.26 (3.82, 4.69)	-0.98 (-2.74, 0.77) p=0.27	21.41 (17.43, 25.40)	4.07 (3.69, 4.45)	-0.09 (-2.14, 1.95) p=0.93
**Diabetes duration (years)**	18.87 (14.11, 23.63)	4.22 (3.79, 4.65)	0.02 (-0.04, 0.09) p=0.48	20.87 (16.51, 25.23)	4.07 (3.69, 4.45)	0.02 (-0.05, 0.09) p=0.55
**Creatinine (µmol/l)**	18.56 (12.27, 24.84)	4.22 (3.78, 4.66)	0.01 (-0.04, 0.07) p=0.67	21.51 (15.49, 27.52)	4.10 (3.71, 4.49)	-0.01 (-0.07, 0.05) p=0.83
**BMI (kg/m²)**	18.70 (12.33, 25.08)	4.21 (3.78, 4.64)	0.03 (-0.15, 0.20) p=0.75	19.05 (12.64, 25.46)	4.07 (3.69, 4.45)	0.09 (-0.10, 0.27) p=0.35

	GOLD study			SILVER study		
**Covariate**	**Intercept**	**Mean glucose**	**Covariate coefficient**	**Intercept**	**Mean glucose**	**Covariate coefficient**
**HCS**	23.25 (15.99, 30.51)	4.16 (3.73, 4.59)	-0.13 (-0.32, 0.07) p=0.20	23.19 (16.29, 30.10)	4.06 (3.67, 4.44)	-0.07 (-0.27, 0.14) p=0.52
**DTSQ**	17.85 (11.55, 24.15)	4.21 (3.78, 4.64)	0.05 (-0.08, 0.19) p=0.46	19.40 (13.13, 25.67)	4.07 (3.69, 4.45)	0.06 (-0.09, 0.22) p=0.43
**C-peptide (nmol/l)**	19.49 (14.79, 24.19)	4.18 (3.74, 4.62)	4.64 (-16.14, 25.41) p=0.66	21.44 (17.08, 25.80)	4.03 (3.64, 4.41)	7.14 (-26.72, 41.00) p=0.68
**Total cholesterol (mmol/l)**	19.89 (14.13, 25.66)	4.21 (3.77, 4.65)	-0.10 (-0.94, 0.75) p=0.82	17.43 (11.75, 23.11)	4.09 (3.70, 4.47)	0.83 (-0.09, 1.76) p=0.076
**LDL cholesterol (mmol/l)***	19.74 (14.65, 24.82)	4.21 (3.77, 4.65)	-0.13 (-1.22, 0.96) p=0.82	19.21 (14.43, 23.99)	4.11 (3.73, 4.50)	0.82 (-0.31, 1.95) p=0.15
**HDL cholesterol (mmol/l)***	19.54 (14.87, 24.21)	4.21 (3.77, 4.65)	-0.06 (-1.19, 1.06) p=0.91	20.43 (16.27, 24.58)	4.07 (3.68, 4.45)	0.87 (-0.32, 2.06) p=0.15
**Triacylglycerols (mmol/l)***	19.49 (14.91, 24.08)	4.21 (3.78, 4.65)	0.04 (-0.62, 0.70) p=0.90	21.22 (17.13, 25.31)	4.10 (3.71, 4.48)	0.27 (-0.51, 1.04) p=0.49
**APOA1 (g/l)***	19.60 (14.72, 24.48)	4.21 (3.78, 4.65)	-0.12 (-1.78, 1.54) p=0.89	19.60 (15.29, 23.91)	4.05 (3.66, 4.43)	1.72 (0.01, 3.43) p=0.048
**APOB (g/l)***	19.70 (15.00, 24.40)	4.20 (3.76, 4.64)	0.30 (-1.12, 1.72) p=0.67	21.62 (17.48, 25.77)	4.09 (3.71, 4.48)	1.23 (-0.35, 2.82) p=0.13
**CRP (mg/l)***	19.73 (15.13, 24.33)	4.17 (3.73, 4.61)	0.19 (-0.11, 0.48) p=0.21	21.01 (16.91, 25.12)	4.10 (3.71, 4.49)	-0.01 (-0.32, 0.30) p=0.96

Statistical analyses were performed using linear mixed effects models. CGM mean and one covariate at a time were included as fixed effects. Subject-specific random intercepts were included as random effects to account for inter-individual deviations from the mean trend and intra-individual correlations in repeated-measures data.
*The covariate was log-transformed prior to analysis. The regression coefficient represents the expected change in HbA_{1c} per 50% increase in the covariate.

Abbreviations: APOA1, apolipoprotein A1; APOB, apolipoprotein B; BMI, body mass index; CGM, continuous glucose monitoring; CI, confidence interval; CRP, C-reactive protein; CV, coefficient of variation; DTSQ, diabetes treatment satisfaction questionnaire; HCS, hypoglycaemia confidence scale; HDL, high density lipoprotein; LDL, low density lipoprotein; MAGE, mean amplitude of glycaemic excursions; SD, standard deviation.

ESM Table 2. Interaction analyses relating HbA_{1c} (mmol/mol) to mean glucose (mmol/l), glycaemic variability measures, time in glycaemic ranges, patient characteristics, and baseline covariates. Results are presented as regression coefficients with 95% confidence intervals. The interaction effect represents the interaction between mean glucose and the covariate.

	GOLD study				SILVER study			
**Covariate**	**Intercept**	**Mean glucose**	**Covariate coefficient**	**Interaction**	**Intercept**	**Mean glucose**	**Covariate coefficient**	**Interaction**
**CGM SD (mmol/l)**	11.30 (-12.08, 34.67)	5.26 (2.95, 7.57)	1.58 (-4.36, 7.52)	-0.22 (-0.77, 0.34) p=0.45	3.83 (-16.03, 23.70)	5.84 (3.85, 7.82)	4.42 (-0.65, 9.49)	-0.44 (-0.92, 0.04) p=0.075
**CGM CV (%)**	45.81 (15.85, 75.76)	2.01 (-0.79, 4.81)	-69.23 (-149.4, 10.89)	5.79 (-1.80, 13.39) p=0.13	7.70 (-13.34, 28.73)	5.37 (3.39, 7.35)	39.16 (-19.39, 97.71)	-3.75 (-9.40, 1.89) p=0.19
**MAGE (mmol/l)**	8.23 (-17.51, 33.98)	5.54 (3.03, 8.06)	1.07 (-1.78, 3.91)	-0.13 (-0.40, 0.14) p=0.34	1.85 (-16.22, 19.92)	6.07 (4.29, 7.86)	2.13 (0.14, 4.11)	-0.22 (-0.41, -0.02) p=0.027
**Percentage of time below range (<3.9 mmol/l)**	24.70 (18.35, 31.05)	3.72 (3.15, 4.30)	-1.75 (-3.19, -0.32)	0.18 (0.02, 0.34) p=0.030	18.95 (13.59, 24.30)	4.25 (3.77, 4.73)	0.31 (-1.00, 1.62)	-0.01 (-0.15, 0.13) p=0.86
**Percentage of time in range (3.9–10.0 mmol/l)**	32.48 (10.72, 54.24)	3.24 (1.77, 4.71)	-0.18 (-0.43, 0.07)	0.01 (-0.01, 0.04) p=0.33	40.41 (22.68, 58.15)	2.95 (1.78, 4.13)	-0.10 (-0.29, 0.09)	-0.01 (-0.03, 0.01) p=0.57
**Percentage of time in target (3.5–7.8 mmol/l)**	27.63 (12.30, 42.97)	3.57 (2.46, 4.69)	-0.18 (-0.47, 0.11)	0.01 (-0.02, 0.05) p=0.44	32.01 (20.01, 44.00)	3.46 (2.60, 4.31)	0.02 (-0.21, 0.26)	-0.02 (-0.05, 0.01) p=0.22
**Percentage of time above range level 2 (>13.9 mmol/l)**	11.71 (-0.71, 24.13)	5.08 (3.55, 6.62)	0.13 (-0.27, 0.53)	-0.02 (-0.04, 0.01) p=0.20	16.37 (5.82, 26.93)	4.67 (3.40, 5.94)	-0.00 (-0.28, 0.27)	-0.01 (-0.02, 0.01) p=0.56
**Female**	17.43 (11.36, 23.50)	4.40 (3.81, 4.99)	4.63 (-4.52, 13.79)	-0.42 (-1.29, 0.45) p=0.34	21.40 (16.16, 26.63)	4.01 (3.50, 4.51)	1.05 (-7.01, 9.11)	0.06 (-0.70, 0.82) p=0.88
**Age (years)**	20.66 (2.57, 38.75)	4.00 (2.27, 5.72)	-0.03 (-0.43, 0.37)	0.01 (-0.03, 0.04) p=0.78	26.41 (11.38, 41.45)	3.46 (2.03, 4.90)	-0.11 (-0.43, 0.20)	0.01 (-0.02, 0.04) p=0.39
**Female < 50 years**	17.46 (12.12, 22.80)	4.43 (3.91, 4.95)	5.16 (-5.11, 15.43)	-0.58 (-1.53, 0.37) p=0.23	21.03 (16.34, 25.73)	4.11 (3.66, 4.56)	1.29 (-7.87, 10.44)	-0.13 (-0.97, 0.71) p=0.76
**Diabetes duration (years)**	21.73 (12.74, 30.72)	3.94 (3.09, 4.79)	-0.11 (-0.49, 0.26)	0.01 (-0.02, 0.05) p=0.46	22.92 (14.91, 30.92)	3.87 (3.09, 4.64)	-0.07 (-0.39, 0.24)	0.01 (-0.02, 0.04) p=0.55
**Creatinine (μmol/l)**	29.23 (6.01, 52.44)	3.22 (1.09, 5.35)	-0.14 (-0.47, 0.18)	0.01 (-0.02, 0.04) p=0.35	33.20 (14.13, 52.28)	2.99 (1.23, 4.75)	-0.17 (-0.43, 0.09)	0.02 (-0.01, 0.04) p=0.20
**BMI (kg/m²)**	26.40 (-4.82, 57.62)	3.49 (0.59, 6.39)	-0.26 (-1.43, 0.91)	0.03 (-0.08, 0.14) p=0.62	48.47 (23.05, 73.90)	1.18 (-1.26, 3.62)	-1.04 (-1.99, -0.08)	0.11 (0.02, 0.20) p=0.019
**HCS**	31.94 (5.13, 58.75)	3.35 (0.90, 5.80)	-0.47 (-1.52, 0.57)	0.03 (-0.06, 0.13) p=0.51	24.73 (2.95, 46.51)	3.91 (1.94, 5.89)	-0.13 (-1.00, 0.74)	0.01 (-0.07, 0.09) p=0.88
**DTSQ**	51.33 (26.16, 76.50)	1.03 (-1.32, 3.39)	-1.04 (-1.84, -0.23)	0.10 (0.03, 0.18) p=0.007	26.77 (3.08, 50.47)	3.36 (1.12, 5.60)	-0.18 (-0.93, 0.58)	0.02 (-0.05, 0.09) p=0.53
**C-peptide (nmol/l)**	21.58 (13.49, 29.67)	3.98 (3.22, 4.74)	-37.04 (-170.23, 96.15)	3.93 (-8.48, 16.35) p=0.53	24.37 (14.87, 33.88)	3.75 (2.85, 4.64)	-57.33 (-246.07, 131.42)	6.19 (-11.64, 24.01) p=0.50

Covariate	GOLD study Intercept	GOLD study Mean glucose	GOLD study Covariate coefficient	GOLD study Interaction	SILVER study Intercept	SILVER study Mean glucose	SILVER study Covariate coefficient	SILVER study Interaction
**Total cholesterol (mmol/l)**	4.76 (-17.55, 27.08)	5.66 (3.55, 7.76)	3.19 (-1.58, 7.95)	-0.31 (-0.76, 0.13) p=0.17	19.42 (-0.09, 38.94)	3.89 (2.04, 5.75)	0.39 (-3.82, 4.60)	0.04 (-0.36, 0.44) p=0.83
**LDL cholesterol (mmol/l)***	16.23 (1.66, 30.80)	4.55 (3.16, 5.95)	1.51 (-4.97, 7.99)	-0.16 (-0.78, 0.46) p=0.61	22.14 (10.01, 34.26)	3.83 (2.70, 4.96)	-0.63 (-6.27, 5.00)	0.14 (-0.39, 0.67) p=0.60
**HDL cholesterol (mmol/l)***	15.15 (6.59, 23.71)	4.63 (3.82, 5.44)	3.41 (-2.47, 9.29)	-0.33 (-0.87, 0.22) p=0.24	19.33 (11.54, 27.11)	4.17 (3.43, 4.91)	1.71 (-3.45, 6.88)	-0.08 (-0.55, 0.40) p=0.74
**Triacylglycerols (mmol/l)***	20.18 (14.67, 25.70)	4.14 (3.61, 4.67)	1.01 (-3.38, 5.40)	-0.09 (-0.52, 0.33) p=0.66	19.76 (14.97, 24.55)	4.24 (3.79, 4.69)	-2.01 (-5.96, 1.95)	0.22 (-0.15, 0.59) p=0.25
**APOA1 (g/l)***	13.39 (2.03, 24.75)	4.79 (3.74, 5.85)	4.73 (-3.58, 13.04)	-0.45 (-1.21, 0.31) p=0.24	18.29 (7.80, 28.78)	4.17 (3.18, 5.16)	2.71 (-4.72, 10.14)	-0.09 (-0.78, 0.59) p=0.79
**APOB (g/l)***	21.37 (15.36, 27.37)	4.04 (3.48, 4.61)	4.22 (-4.72, 13.16)	-0.38 (-1.24, 0.48) p=0.38	19.83 (14.40, 25.25)	4.27 (3.76, 4.78)	-2.54 (-10.02, 4.95)	0.37 (-0.34, 1.08) p=0.31
**CRP (mg/l)***	18.16 (13.25, 23.08)	4.33 (3.85, 4.80)	1.65 (0.05, 3.25)	-0.14 (-0.29, 0.01) p=0.069	20.75 (16.30, 25.21)	4.13 (3.70, 4.56)	0.18 (-1.12, 1.48)	-0.02 (-0.14, 0.10) p=0.77

Statistical analyses were performed using linear mixed effects models. CGM mean, covariate, and covariate with CGM mean interaction were included as fixed effects. Subject-specific random intercepts were included as random effects to account for inter-individual deviations from the mean trend and intra-individual correlations in repeated-measures data.
*The covariate was log-transformed prior to analysis. The regression coefficient represents the expected change in HbA_{1c} per 50% increase in the covariate.

Abbreviations: APOA1, apolipoprotein A1; APOB, apolipoprotein B; BMI, body mass index; CGM, continuous glucose monitoring; CI, confidence interval; CRP, C-reactive protein; CV, coefficient of variation; DTSQ, diabetes treatment satisfaction questionnaire; HCS, hypoglycaemia confidence scale; HDL, high density lipoprotein; LDL, low density lipoprotein; MAGE, mean amplitude of glycaemic excursions; SD, standard deviation.

ESM Table 3. Linear mixed-effects regression models relating HbA_{1c} (mmol/mol) to time in range (TIR, 3.9–10.0 mmol/l), glycaemic variability measures, time in glycaemic ranges, patient characteristics, and baseline covariates. Results are presented as regression coefficients with 95% confidence intervals.

	GOLD study (n=144)			SILVER study (n=98)		
**Covariate**	**Intercept**	**Percentage TIR**	**Covariate coefficient**	**Intercept**	**Percentage TIR**	**Covariate coefficient**
**CGM Mean (mmol/l)**	30.87 (9.35, 52.39)	-0.09 (-0.24, 0.07)	3.51 (2.14, 4.88) p<0.001	40.53 (22.83, 58.24)	-0.14 (-0.27, -0.01)	2.88 (1.74, 4.01) p<0.001
**CGM SD (mmol/l)**	86.29 (78.56, 94.01)	-0.47 (-0.54, -0.41)	-0.13 (-1.50, 1.24) p=0.85	86.76 (80.19, 93.33)	-0.45 (-0.51, -0.40)	-0.36 (-1.55, 0.83) p=0.56
**CGM CV (%) #**	91.74 (86.36, 97.13)	-0.45 (-0.51, -0.40)	-18.33 (-32.5, -4.14) p=0.012	89.16 (84.68, 93.64)	-0.43 (-0.48, -0.39)	-12.67 (-24.3, -1.09) p=0.032
**MAGE (mmol/l)**	87.99 (80.34, 95.65)	-0.48 (-0.54, -0.42)	-0.20 (-0.80, 0.40) p=0.52	89.05 (84.27, 93.84)	-0.46 (-0.51, -0.42)	-0.35 (-0.70, 0.00) p=0.053
**Percentage of time below range (<3.9 mmol/l)**	85.03 (82.55, 87.52)	-0.42 (-0.47, -0.36)	-0.64 (-0.89, -0.38) p<0.001	84.95 (82.71, 87.20)	-0.42 (-0.47, -0.38)	-0.29 (-0.52, -0.07) p=0.012
**Percentage of time in target (3.5–7.8 mmol/l)**	84.27 (81.29, 87.24)	-0.35 (-0.49, -0.20)	-0.16 (-0.34, 0.02) p=0.077	84.05 (81.62, 86.47)	-0.34 (-0.45, -0.24)	-0.13 (-0.27, -0.00) p=0.046
**Percentage of time above range level 2 (>13.9 mmol/l)**	73.51 (65.17, 81.85)	-0.30 (-0.42, -0.18)	0.20 (0.07, 0.34) p=0.003	75.65 (68.63, 82.68)	-0.31 (-0.42, -0.21)	0.15 (0.04, 0.27) p=0.007
**Female**	85.25 (82.47, 88.03)	-0.47 (-0.52, -0.42)	0.54 (-1.07, 2.14) p=0.51	84.19 (81.73, 86.65)	-0.44 (-0.48, -0.39)	1.43 (-0.41, 3.27) p=0.13
**Age (years)**	83.97 (80.53, 87.41)	-0.48 (-0.53, -0.42)	0.04 (-0.02, 0.11) p=0.15	83.26 (79.46, 87.07)	-0.44 (-0.49, -0.40)	0.04 (-0.03, 0.11) p=0.29
**Female < 50 years**	86.06 (83.31, 88.81)	-0.47 (-0.53, -0.42)	-0.86 (-2.67, 0.94) p=0.35	84.98 (82.56, 87.39)	-0.44 (-0.49, -0.40)	-0.22 (-2.38, 1.95) p=0.84
**Diabetes duration (years)**	85.08 (82.16, 88.01)	-0.47 (-0.52, -0.42)	0.02 (-0.04, 0.09) p=0.46	84.24 (81.40, 87.09)	-0.44 (-0.49, -0.40)	0.03 (-0.05, 0.11) p=0.44
**Creatinine (µmol/l)**	84.96 (80.32, 89.60)	-0.47 (-0.53, -0.42)	0.01 (-0.05, 0.07) p=0.68	84.91 (79.86, 89.96)	-0.45 (-0.49, -0.40)	0.00 (-0.06, 0.07) p=0.89

Covariate	GOLD study (n=144)			SILVER study (n=98)		
	Intercept	Percentage TIR	Covariate coefficient	Intercept	Percentage TIR	Covariate coefficient
**BMI (kg/m²)**	83.01 (77.52, 88.50)	-0.47 (-0.52, -0.42)	0.09 (-0.08, 0.27) p=0.29	81.77 (76.09, 87.45)	-0.44 (-0.49, -0.40)	0.12 (-0.08, 0.32) p=0.23
**HCS**	88.25 (82.88, 93.61)	-0.46 (-0.52, -0.41)	-0.11 (-0.32, 0.09) p=0.26	87.08 (81.40, 92.75)	-0.44 (-0.49, -0.40)	-0.09 (-0.30, 0.13) p=0.42
**DTSQ**	84.39 (79.45, 89.32)	-0.47 (-0.52, -0.42)	0.04 (-0.10, 0.18) p=0.58	83.00 (77.48, 88.52)	-0.44 (-0.49, -0.40)	0.06 (-0.10, 0.23) p=0.44
**C-peptide (nmol/l)**	85.08 (82.26, 87.90)	-0.46 (-0.52, -0.41)	3.45 (-17.75, 24.65) p=0.75	84.22 (81.43, 87.01)	-0.44 (-0.48, -0.39)	8.03 (-27.51, 43.57) p=0.66
**Total cholesterol (mmol/l)**	85.57 (80.72, 90.41)	-0.47 (-0.52, -0.42)	0.04 (-0.83, 0.91) p=0.93	80.64 (75.66, 85.61)	-0.45 (-0.49, -0.40)	1.02 (0.04, 1.99) p=0.041
**LDL cholesterol (mmol/l)***	86.25 (82.59, 89.91)	-0.47 (-0.53, -0.42)	-0.21 (-1.33, 0.90) p=0.71	83.45 (80.10, 86.80)	-0.45 (-0.50, -0.41)	0.88 (-0.31, 2.08) p=0.15
**HDL cholesterol (mmol/l)***	85.18 (82.24, 88.12)	-0.47 (-0.52, -0.42)	0.51 (-0.64, 1.66) p=0.38	83.70 (80.89, 86.50)	-0.45 (-0.49, -0.40)	1.22 (-0.03, 2.47) p=0.055
**Triacylglycerols (mmol/l)***	85.79 (83.12, 88.45)	-0.47 (-0.52, -0.42)	0.06 (-0.62, 0.74) p=0.86	85.45 (83.05, 87.84)	-0.45 (-0.49, -0.40)	0.37 (-0.45, 1.19) p=0.37
**APOA1 (g/l)***	84.93 (81.62, 88.24)	-0.47 (-0.52, -0.42)	0.70 (-1.00, 2.39) p=0.42	82.16 (78.91, 85.41)	-0.44 (-0.49, -0.40)	2.39 (0.61, 4.16) p=0.009
**APOB (g/l)***	85.84 (83.18, 88.50)	-0.47 (-0.52, -0.42)	0.37 (-1.09, 1.82) p=0.62	85.84 (83.41, 88.28)	-0.45 (-0.49, -0.40)	1.50 (-0.18, 3.18) p=0.080
**CRP (mg/l)***	85.18 (82.50, 87.85)	-0.47 (-0.52, -0.41)	0.31 (0.01, 0.61) p=0.043	85.08 (82.68, 87.48)	-0.45 (-0.49, -0.40)	0.10 (-0.23, 0.42) p=0.56

Statistical analyses were performed using linear mixed effects models. Percentage time in range and one covariate at a time were included as fixed effects. Subject-specific random intercepts were included as random effects to account for inter-individual deviations from the mean trend and intra-individual correlations in repeated-measures data.
*The covariate was log-transformed prior to analysis. The regression coefficient represents the expected change in HbA_{1c} per 50% increase in the covariate.
#Not significant when accounting for time below range (<3.9 mmol/l) and time above range level 2 (>13.9 mmol/l).
Abbreviations: APOA1, apolipoprotein A1; APOB, apolipoprotein B; BMI, body mass index; CGM, continuous glucose monitoring; CI, confidence interval; CRP, C-reactive protein; CV, coefficient of variation; DTSQ, diabetes treatment satisfaction questionnaire; HCS, hypoglycaemia confidence scale; HDL, high density lipoprotein; LDL, low density lipoprotein; MAGE, mean amplitude of glycaemic excursions; SD, standard deviation; TIR, time in range.

ESM Table 4. Interaction analyses relating HbA_{1c} (mmol/mol) to time in range (TIR, 3.9–10.0 mmol/l), glycaemic variability measures, time in glycaemic ranges, patient characteristics, and baseline covariates. Results are presented as regression coefficients with 95% confidence intervals. The interaction effect represents the interaction between TIR and the covariate.

	GOLD study (n=144)				SILVER study (n=98)			
**Covariate**	**Intercept**	**Percentage TIR**	**Covariate coefficient**	**Interaction**	**Intercept**	**Percentage TIR**	**Covariate coefficient**	**Interaction**
**CGM Mean (mmol/l)**	32.48 (10.72, 54.24)	-0.18 (-0.43, 0.07)	3.24 (1.77, 4.71)	0.01 (-0.01, 0.04) p=0.33	40.41 (22.68, 58.15)	-0.10 (-0.29, 0.09)	2.95 (1.78, 4.13)	-0.01 (-0.03, 0.01) p=0.57
**CGM SD (mmol/l) #**	68.31 (54.28, 82.33)	-0.09 (-0.35, 0.17)	4.56 (1.19, 7.94)	-0.10 (-0.17, -0.03) p=0.003	75.23 (63.07, 87.40)	-0.22 (-0.43, -0.00)	2.69 (-0.27, 5.65)	-0.06 (-0.12, -0.01) p=0.028
**CGM CV (%) #**	69.03 (52.16, 85.89)	0.04 (-0.31, 0.40)	46.18 (-1.54, 93.91)	-1.39 (-2.38, -0.41) p=0.006	92.82 (80.80, 104.84)	-0.51 (-0.75, -0.28)	-23.55 (-58.6, 11.5)	0.23 (-0.45, 0.90) p=0.51
**MAGE (mmol/l)**	70.89 (55.80, 85.98)	-0.12 (-0.40, 0.17)	1.74 (0.14, 3.33)	-0.04 (-0.08, -0.01) p=0.011	83.84 (74.09, 93.60)	-0.35 (-0.54, -0.16)	0.26 (-0.80, 1.32)	-0.01 (-0.04, 0.01) p=0.23
**Percentage of time below range (<3.9 mmol/l)**	83.80 (80.58, 87.01)	-0.39 (-0.46, -0.32)	-0.02 (-1.08, 1.04)	-0.01 (-0.03, 0.01) p=0.24	86.03 (83.34, 88.73)	-0.45 (-0.50, -0.39)	-0.87 (-1.69, -0.05)	0.01 (-0.00, 0.03) p=0.15
**Percentage of time in target (3.5–7.8 mmol/l)**	87.71 (82.94, 92.48)	-0.40 (-0.55, -0.24)	-0.36 (-0.64, -0.08)	0.00 (-0.00, 0.01) p=0.071	88.22 (84.48, 91.97)	-0.40 (-0.51, -0.29)	-0.41 (-0.64, -0.18)	0.00 (0.00, 0.01) p=0.005
**Percentage of time above range level 2 (>13.9 mmol/l)**	73.38 (64.95, 81.81)	-0.30 (-0.42, -0.18)	0.19 (0.04, 0.35)	0.00 (-0.00, 0.00) p=0.81	76.76 (69.60, 83.92)	-0.32 (-0.42, -0.21)	0.19 (0.07, 0.31)	-0.00 (-0.00, 0.00) p=0.13
**Female**	85.83 (82.23, 89.44)	-0.48 (-0.55, -0.41)	-0.70 (-5.91, 4.51)	0.03 (-0.08, 0.13) p=0.62	84.53 (81.38, 87.68)	-0.45 (-0.50, -0.39)	0.69 (-3.89, 5.27)	0.02 (-0.07, 0.10) p=0.73
**Age (years)**	88.25 (78.53, 97.97)	-0.57 (-0.76, -0.37)	-0.05 (-0.27, 0.16)	0.00 (-0.00, 0.01) p=0.36	80.46 (71.94, 88.97)	-0.39 (-0.55, -0.23)	0.10 (-0.08, 0.28)	-0.00 (-0.00, 0.00) p=0.47
**Female < 50 years**	86.17 (83.01, 89.33)	-0.48 (-0.54, -0.42)	-1.25 (-6.90, 4.40)	0.01 (-0.11, 0.13) p=0.89	84.89 (82.17, 87.61)	-0.44 (-0.49, -0.39)	0.09 (-5.03, 5.21)	-0.01 (-0.11, 0.10) p=0.90
**Diabetes duration (years)**	84.02 (78.94, 89.11)	-0.45 (-0.55, -0.35)	0.08 (-0.14, 0.29)	-0.00 (-0.01, 0.00) p=0.62	84.37 (79.32, 89.42)	-0.45 (-0.54, -0.35)	0.02 (-0.18, 0.22)	0.00 (-0.00, 0.00) p=0.95
**Creatinine (µmol/l)**	82.25 (69.29, 95.21)	-0.41 (-0.69, -0.14)	0.05 (-0.13, 0.23)	-0.00 (-0.00, 0.00) p=0.66	82.29 (71.45, 93.13)	-0.39 (-0.60, -0.19)	0.04 (-0.11, 0.19)	-0.00 (-0.00, 0.00) p=0.59
**BMI (kg/m²)**	82.16 (65.09, 99.23)	-0.45 (-0.81, -0.09)	0.13 (-0.51, 0.76)	-0.00 (-0.01, 0.01) p=0.92	70.77 (56.69, 84.84)	-0.22 (-0.48, 0.04)	0.54 (0.01, 1.06)	-0.01 (-0.02, 0.00) p=0.094
**HCS**	83.75 (69.07, 98.43)	-0.36 (-0.67, -0.06)	0.07 (-0.52, 0.65)	-0.00 (-0.02, 0.01) p=0.52	87.88 (75.75, 100.00)	-0.46 (-0.70, -0.22)	-0.12 (-0.61, 0.37)	0.00 (-0.01, 0.01) p=0.88
**DTSQ**	76.00 (61.76, 90.25)	-0.29 (-0.58, -0.00)	0.31 (-0.14, 0.76)	-0.01 (-0.01, 0.00) p=0.22	83.24 (69.74, 96.74)	-0.45 (-0.71, -0.19)	0.06 (-0.37, 0.48)	0.00 (-0.01, 0.01) p=0.97

Covariate	GOLD study (n=144)				SILVER study (n=98)			
	Intercept	Percentage TIR	Covariate coefficient	Interaction	Intercept	Percentage TIR	Covariate coefficient	Interaction
**C-peptide (nmol/l)**	85.76 (81.65, 89.87)	-0.48 (-0.56, -0.40)	-8.98 (-68.25, 50.29)	0.28 (-0.96, 1.51) p=0.66	83.27 (78.27, 88.27)	-0.42 (-0.51, -0.32)	28.69 (-67.98, 125.37)	-0.43 (-2.29, 1.43) p=0.65
**Total cholesterol (mmol/l)**	96.88 (84.04, 109.73)	-0.71 (-0.97, -0.45)	-2.40 (-5.10, 0.31)	0.05 (-0.00, 0.11) p=0.062	75.92 (64.38, 87.46)	-0.35 (-0.57, -0.14)	2.06 (-0.45, 4.58)	-0.02 (-0.07, 0.03) p=0.37
**LDL cholesterol (mmol/l)***	91.34 (83.01, 99.68)	-0.58 (-0.74, -0.41)	-2.57 (-6.19, 1.05)	0.05 (-0.02, 0.12) p=0.18	82.00 (75.52, 88.48)	-0.42 (-0.55, -0.29)	1.61 (-1.42, 4.64)	-0.02 (-0.07, 0.04) p=0.61
**HDL cholesterol (mmol/l)***	86.92 (82.03, 91.82)	-0.51 (-0.61, -0.41)	-0.86 (-4.20, 2.48)	0.03 (-0.04, 0.10) p=0.39	81.57 (77.17, 85.97)	-0.40 (-0.48, -0.32)	2.91 (-0.07, 5.89)	-0.04 (-0.09, 0.02) p=0.22
**Triacylglycerols (mmol/l)***	85.08 (82.08, 88.09)	-0.46 (-0.52, -0.40)	-1.12 (-3.55, 1.31)	0.02 (-0.02, 0.07) p=0.32	85.59 (82.95, 88.22)	-0.45 (-0.50, -0.40)	0.62 (-1.50, 2.75)	-0.01 (-0.05, 0.04) p=0.80
**APOA1 (g/l)***	88.26 (81.74, 94.77)	-0.55 (-0.68, -0.41)	-1.86 (-6.57, 2.85)	0.06 (-0.04, 0.15) p=0.25	78.79 (72.77, 84.81)	-0.37 (-0.49, -0.26)	5.00 (0.69, 9.31)	-0.06 (-0.14, 0.03) p=0.19
**APOB (g/l)***	84.75 (81.51, 87.99)	-0.45 (-0.51, -0.38)	-2.45 (-7.52, 2.61)	0.06 (-0.04, 0.16) p=0.25	86.91 (83.83, 89.98)	-0.47 (-0.53, -0.41)	3.78 (-0.54, 8.09)	-0.05 (-0.13, 0.04) p=0.26
**CRP (mg/l)***	85.92 (83.01, 88.82)	-0.48 (-0.54, -0.42)	-0.27 (-1.18, 0.63)	0.01 (-0.01, 0.03) p=0.18	84.73 (82.16, 87.31)	-0.44 (-0.49, -0.39)	0.36 (-0.42, 1.14)	-0.01 (-0.02, 0.01) p=0.46

Statistical analyses were performed using linear mixed effects models. Percentage time in range, covariate, and covariate with CGM mean interaction were included as fixed effects. Subject-specific random intercepts were included as random effects to account for inter-individual deviations from the mean trend and intra-individual correlations in repeated-measures data.

*The covariate was log-transformed prior to analysis. The regression coefficient represents the expected change in HbA_{1c} per 50% increase in the covariate.

#Not significant when accounting for time below range (<3.9 mmol/l) and time above range level 2 (>13.9 mmol/l).

Abbreviations: APOA1, apolipoprotein A1; APOB, apolipoprotein B; BMI, body mass index; CGM, continuous glucose monitoring; CI, confidence interval; CRP, C-reactive protein; CV, coefficient of variation; DTSQ, diabetes treatment satisfaction questionnaire; HCS, hypoglycaemia confidence scale; HDL, high density lipoprotein; LDL, low density lipoprotein; MAGE, mean amplitude of glycaemic excursions; SD, standard deviation; TIR, time in range.

ESM Figure 1. Non-linear association between HbA_{1c} and the distribution of glucose values derived on data from the GOLD trial (*n*=144). The estimated HbA_{1c} (eA_{1c}) is expressed as a weighted sum (integral) of the individual glucose values (x) with weight function $\beta(x)$. $dP(x)$ is a probability measure, i.e., the relative frequency or density of glucose values. A linear weight function corresponds to a linear relationship between HbA_{1c} and MG (cf. Figure 1). The weight function was determined by best subset selection (minimise Akaike's Information Criterion) on all fractional polynomial models with up to three parameters.

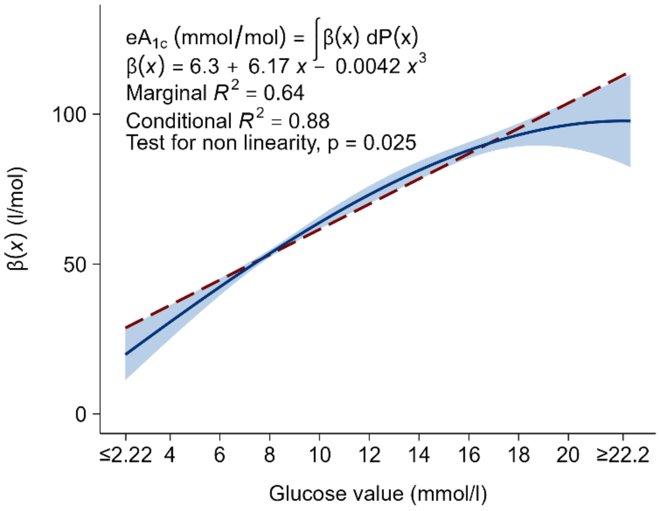

ESM Figure 2. Non-linear association between HbA_{1c} and the distribution of glucose values validated on data from the SILVER trial (*n*=98). The estimated HbA_{1c} (eA_{1c}) is expressed as a weighted sum (integral) of the individual glucose values (*x*) with weight function *β(x)*. *dP(x)* is a probability measure, i.e., the relative frequency or density of glucose values. A linear weight function corresponds to a linear relationship between HbA_{1c} and MG (cf. Figure 1). The same fractional polynomial weight function as in Figure S1 was used and re-fitted on data from the SILVER study.

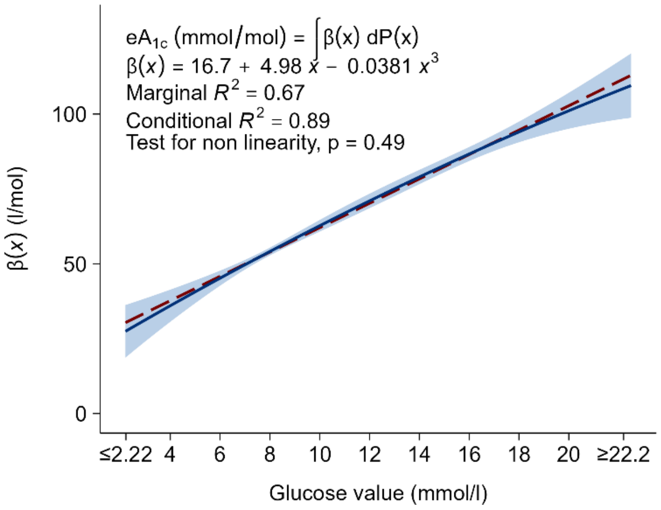

ESM Figure 3. Estimated mean glucose (MG) at a given level of TIR (3.9–10.0 mmol/l) and TBR (<3.9 mmol/l).

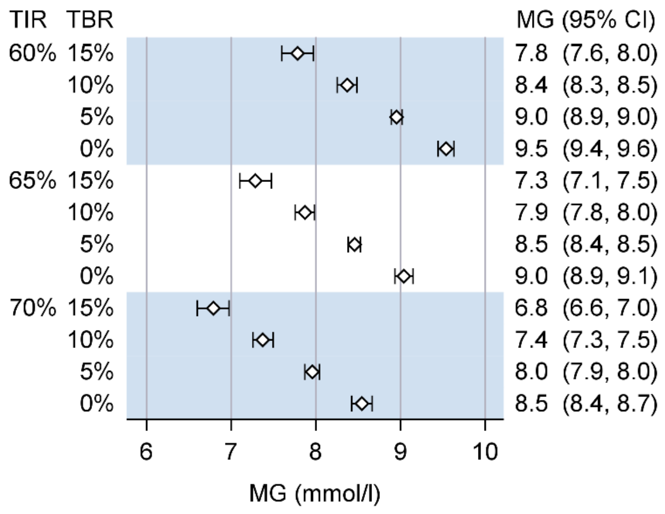

Supplement: Supplementary file 1 — Supplementary file1 (PDF 415 KB) [file 125_2024_6151_MOESM1_ESM.pdf]
